# Neurons in Primary Motor Cortex Encode External Perturbations during an Orientation Reaching Task

**DOI:** 10.3390/brainsci11091125

**Published:** 2021-08-25

**Authors:** Yan Ma, Weiming Sun, Nanrun Zhou, Jiping He, Chaolin Ma

**Affiliations:** 1 Department of Computer Science and Engineering, School of Information Engineering, Nanchang University, Nanchang 330031, China; ma.yan1991@email.ncu.edu.cn (Y.M.); Nrzhou@ncu.edu.cn (N.Z.); 2 School of Biological and Health Systems Engineering, Arizona State University, Tempe, AZ 85287, USA; 3 Institutes of Life Science, Nanchang University, Nanchang 330031, China; sunweiming@email.ncu.edu.cn; 4 School of Life Science, Nanchang University, Nanchang 330031, China

**Keywords:** primary motor cortex, neuronal ensemble recording, external perturbation of orientation, reaching orientation task, monkey

## Abstract

When confronting an abrupt external perturbation force during movement, subjects continuously adjust their behaviors to adapt to changes. Such adaptation is of great importance for realizing flexible motor control in varied environments, but the potential cortical neuronal mechanisms behind it have not yet been elucidated. Aiming to reveal potential neural control system compensation for external disturbances, we applied an external orientation perturbation while monkeys performed an orientation reaching task and simultaneously recorded the neural activity in the primary motor cortex (M1). We found that a subpopulation of neurons in the primary motor cortex specially created a time-locked activity in response to a “go” signal in the adaptation phase of the impending orientation perturbation and did not react to a “go” signal under the normal task condition without perturbation. Such neuronal activity was amplified as the alteration was processed and retained in the extinction phase; then, the activity gradually faded out. The increases in activity during the adaptation to the orientation perturbation may prepare the system for the impending response. Our work provides important evidence for understanding how the motor cortex responds to external perturbations and should advance research about the neurophysiological mechanisms underlying motor learning and adaptation.

## 1. Introduction

When confronting an abrupt external perturbing force during movement, subjects constantly adjust their behavior to correspond to instant changes. Such alteration is essential for flexible motor control in varied environments [1], but it is still not clear what strategies the brain utilizes to solve such learning and adaptation issues. The computational study of motor control is concerned with the association of motor commands and sensory feedback signals. Generally speaking, feedback control aiming to eliminate the inconsistency between expected movement and output is often considered to be time consuming and inefficient because the abruptness of such perturbation means that it occurs within a very limited time. In such conditions, a feedforward control mechanism would play an important role in improving the ability of the subjects to adapt. Through continuous motor learning, subjects can make various predictions and preparations for an approaching perturbation. To elucidate the basis of adaptation in initialization, development, and extinction on a neurophysiological level, an exploration of associated neuron ensembles in various cortical regions is significant.

Previous studies have investigated the innervation relationships between neuronal activity patterns from various brain structures and different behaviors [2,3,4]. It has been reported that primary motor cortex (M1) neuronal signals can be used to predict the direction and velocity of arm movements [5,6], based on which a brain–machine interface was developed to help those who suffer from paralysis. However, such systems still present a series of issues regarding the flexibility and robustness of control. A practical requirement for them is that they must be able to compensate for disturbances interfering with normal operations. Understanding what kind of strategies the brain utilizes to solve these issues is helpful for the realization of such aims, but the details still require extensive investigation. Traditionally, reactions to targeted perturbations have delivered a good panel for control strategies for motor systems [7,8,9,10], and perturbations have generally led to a number of meaningful insights into control issues. One of the examples would be the reflex responses that maintain an upright stance in the face of sudden and unpredictable translations of a support surface, including both spinal and long loop reflexes [11]. An additional anticipatory response is developed if a subject receives prior information about an upcoming perturbation; this response can facilitate additional effective control of the perturbation [12]. In addition, anticipation also affects directly voluntary activities, such as reaching [13] and ball catching [14,15]. Our previous study showed that human subjects adopted a feedforward control strategy to predict a target orientation perturbation when the perturbation information was predictable [16].

Our perturbation studies on human subjects showed that a variation in target orientation during prehension leads to an alteration of the hand and arm orientation for grasping. The time to react to a perturbation in the first trial was about 200 ms, which allowed re-planning the execution of orientation. Orientation trajectory alterations were found in repeat perturbation trials but not in random perturbation trials. In repeat perturbation trials, the perturbation reaction time dropped dramatically, which showed that a predictive control strategy was adopted by the movement system. The motor system was capable of remodeling the target orientation while it was in motion with random perturbations. While, during repeat perturbations, monkeys were capable of anticipating perturbations and plotting movements to a non-presented target, their perturbation reaction times dramatically dropped, and the cue reaction time increased, suggesting an extended activation period for an intensified plan.

Previous research in our lab that observed changes in cortical neuronal activities over a period of weeks and months while animals learned to overcome either predictable or unpredictable perturbations during arm reaching movements showed that we can investigate the adaptation process and sensory feedback control in the cortical neuronal circuitry [1,17]. In this work, we built upon our previous findings by studying the control issues at the upper command level. We investigated the changes in the activity patterns of single motor cortical neurons in response to sudden changes in target orientation during 3D reach-to-grasp movements. We tested movements amongst targets with fixed orientations, targets with randomly perturbed orientations, and targets with predictably perturbed orientations. Our focus was on how neural commands and coding were affected by different perturbation conditions. The timing and magnitude of the responses were examined to identify variations in neural activity that could be connected to prediction or online control. A predictable perturbation would present the neural control system with the option of altering the control strategy and updating the internal perturbation model. By contrast, an unpredictable perturbation would prevent alteration and force the central nervous system to select feedback control.

One primary hypothesis regarding the experiments is that the monkey would develop a predictive, feed-forward control strategy to deal with it as a new task during the repeat perturbation. When the features of a specific perturbation are known, the central nervous system (CNS) shifts its control strategy from visual feedback to predominantly feed-forward. This hypothesis posits that changes in the neuronal activity patterns of the primary cortex for controlling reach-to-grasp tasks under different perturbation conditions are not critically dependent on information from visual feedback, although they may correlate with intended movement directions and perturbation directions. This study is significant for the understanding of the control strategy and adaptation, a development reflected in the activity patterns of neuronal populations in the motor cortices when the cortical control system learns a new task or overcomes a perturbation. This research will improve our understanding of the cortical control of hand orientation in prehension movements and presents data useful for developing a robust extraction algorithm. The data could also be used to decode neural signals to control a neuro-prosthetic device under the real-life situation of reach to grasp.

## 2. Materials and Methods

### 2.1. Subjects

To reveal the neural control system compensating for external disturbances, our work collected data from two 4–6-year-old male rhesus monkeys (Macaque Mulatta). The experiments complied with the ethical guidelines for the use of laboratory animals and the NIH policy on Humane Care. In addition, all the procedures were approved by the Institutional Animal Care and Use Committee of Arizona State University in the United States.

### 2.2. Surgery and Data Collection Procedure

In the surgery, the monkey was anesthetized with ketamine (8 mg/kg) initially and then maintained narcotism with Isoflurane (1.5~2.0%). A headpiece was surgically mounted on their skull for head restraint firstly. At the second step, a square 20 mm × 23 mm chamber was mounted on the M1 of the left hemisphere head to give access to record neuronal spikes. And then, the two monkeys were taken back to their room to recovery and were given full courses of Oxytetracycline (20 mg/kg) and Buprenorphine (10 ug/kg).

**Figure 1:**
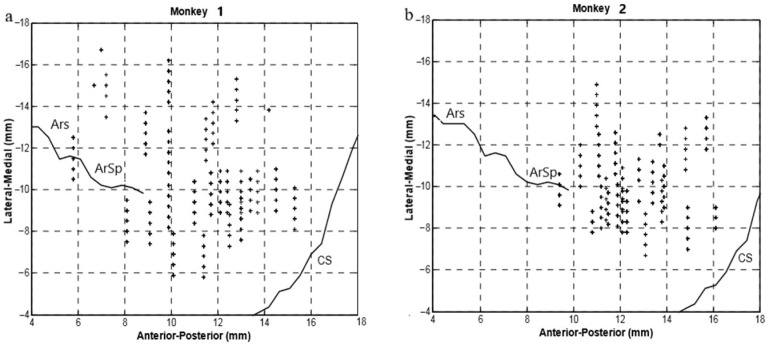
Top view of the electrode penetration locations in the chamber’s coordinates (left hemispheres). (**a**) for Monkey 1; (**b**) for Monkey 2. Each cross represents an electrode penetration. ArS: arcuate sulcus; ArSp: arcuate sulci spur; CS: central sulcus (the brain landmarks were added based on stereotaxic coordinates).

After recovery from the above surgery, the monkeys were prepared for multiple channel single neuron recordings, respectively. A multi-electrode micro-drive (Thomas Recording, Giessen, Germany) was used to insert 5 independently controllable microelectrodes into the M1 hand and arm area through the dura. Every electrode made one penetration per day. The recorded depth at every puncture was zero at the dura. The coordinates of every electrode were precisely calculated and recorded (Figure 1). When we adjusted the electrodes, the system provided the recording depth of every electrode accordingly, and the depth was recorded if we detected a neuron. We monitored a huge number of neurons since the experiment included several blocks every day. Neuronal signals were recorded using the Plexon Multichannel Acquisition Processor System (Plexon, Inc., Dallas, TX, USA) at a sampling frequency of 40 kHz/channel. Putative neuronal units were isolated using Offline Sorter (Plexon, Inc., Dallas, TX, USA) software for all channels with the methods of template matching combined with the principal component. Each channel discriminated up to four units. It should be noted that due to the limitations of our experimental techniques, the characteristics of the neurons recorded, such as the type, have not been fully specified.

Figure 1 shows the locations of the electrode penetrations of the two monkeys and the major brain landmarks in their chamber’s coordinates. Before the cortical signal recording, the location relative to the chamber and its depth relative to the dura were recorded for every neuron. In the surgery, the chamber locations in the stereotaxic coordinates were measured. Then, the calculation was performed based on the matrix coordinates of the rotation from the chamber to the stereotaxic point. Finally, we were able to convert the major landmark coordinates (ArS, CS, and ArSp) in the stereotaxic coordinates into those in the chamber coordinates. The location of the recorded neurons covered the hand representation area of the M1. All the isolated neurons were recorded regardless of their activity during the task.

### 2.3. Behavioral Apparatus

**Figure 2:**
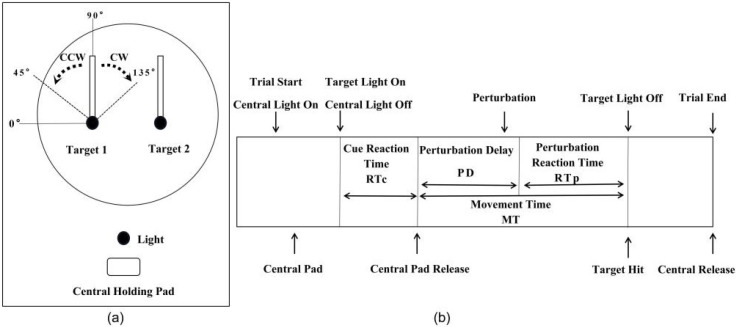
The experimental apparatus used in the study and the sequence of events for the reach-to-grasp task. (**a**) The front view of the experimental apparatus: two targets, central pad, lights, and the target orientation definition. (**b**) The sequence of events for the reach-to-grasp task and the trial epochs. Cue reaction time (RTc), movement time (MT), perturbation delay (PD), and perturbation reaction time (RTp). Cue reaction time is the time from target light coming on to the initiation of reach, indicated by the release of the central holding pad. The movement time is the duration from the release of the holding pad to the grasp of the target. The perturbation delay is the time from the release of the holding pad to perturbation onset. The perturbation reaction time is the time from the perturbation onset to the instant when there was a detectable deviation in the trajectory of the hand orientation to accommodate the new target orientation. The target holding time (THT) was defined as the time from target hit to target release. The center holding time (CHT), was defined as the time from central holding pad hit to target release.

To investigate the performance of the reach-to-grasp task, we designed and constructed an apparatus in which the target orientation could be altered to perturb the planning task (Figure 2a). The apparatus is described in our previous paper in Neuroscience Bulletin [18], which consists of a central holding pad and a frontal plane with two rectangular targets. The central holding pad serves as the initial position. The two rectangular targets are located at roughly shoulder height in the frontal plane. Each target is tailored with sensors indicating successful touching on both sides. To ensure that the perturbation task is a planned prehension task, each target is directly connected to a 1100°/s rotatable programmable servo motor. Thus, the alteration of the target orientation is possible. In addition, the movement distance between the front panel and the center holding pad can be adjusted according to the length of the monkey’s upper limb.

### 2.4. Task and Experiment

The monkey was seated on an immobilized chair in front of the apparatus. While maintaining a steady trunk position, the subjects were instructed to reach and grasp the indicated target using the right hand. Initially, the subjects began each trial by placing their fingers on the central holding pad, and this is what we called a holding phase (Figure 2a). After a 300 ms holding phase, one of the target lights would be lit and cueing the subject to reach for the corresponding target and make the grasp. The sensors were triggered and recorded a successful trial once the subject firmly grasped the target. Alternatively, it recorded a failed trial when the allowed movement time expired. In either case, the target light went off afterward. Finally, we started the next trial by returning the hand to the holding pad. The trial epochs and the sequence of the events are demonstrated in Figure 2b.

Perturbation was defined in this study as a target orientation change of 45° clockwise (CW) or 45° counterclockwise (CCW) 70 ms after the release of the central pad (movement onset). About 130 ms after the initiation of the reaching movement in the perturbation trials, the target orientation changed. The target light was off after the subject made the grip or the allowed time expired. There were four sets of trials for each testing block. Table 1 is a clear classification of the target orientation conditions in different sets. The first set consisted of 90 unperturbed trials. It required the monkey to reach for and grasp one of the two targets, either left or right-oriented at three different angles: 45°, 90°, and 135°. The target orientation changed in a pseudo-random order. It was set to ensure equal probability for every 6 successful trials, which comprised three trials for each target. In addition, both targets were oriented in parallel, and their target lights were seated in a pseudo-random order with equal probability. The last three sets were perturbation trials for target orientation in which the target was either randomly (2nd set) or repeatedly (3rd and 4th sets) rotated from its initial 90° orientation. The randomly perturbed set consisted of the random combination of 1/3 unperturbed and 2/3 perturbed trials. The targets were randomly perturbed 45° CW or 45° CCW, or unperturbed, where the target would stay at 90°. In the 3rd set, both targets were always perturbed at 45° CW. In the 4th set, both targets were repeatedly perturbed at 45° CCW. The occurrence sequence with respect to Set 3 and Set 4 was random. Each block, including 4 sets, was repeated 1–6 times per day depending on the motivation of the subjects.

In the experiment, an optical motion capture system (OPTOTRAK, Northern Digital Inc., Waterloo, ON, Canada) recorded all the hand and arm movements. Three infrared emitting diodes with a sampling rate of 100 Hz were placed on both the hands and forearms of the subjects. Hand orientation trajectories were reconstructed with the markers’ data. Single unit neural signals were collected during each experimental session simultaneously.

### 2.5. Cortical Neuron Discrimination

To determine the spiking characteristics of each neuron across normal and perturbation sessions, signals from the same neurons had to be recorded persistently throughout each block. Although the electrode positions were fixed during each block, it was still difficult to guarantee that the same cells were being recorded from start to finish. However, by examining the waveforms and discharge profiles of individual cells from recordings made on different sets within each block, it was shown that the neural activity was reasonably stable within a whole recording block. To isolate the activity of specific cells, waveform discrimination parameters were adjusted at the beginning of each recording block. Those parameters were kept the same within each recording block (including the 4 sets of movements, for a total of 240 trials). To ensure consistent recordings, careful attention was paid to the shape of the extracellular potentials being recorded by the electrodes within each block. If any changes in the waveform of a unit were observed, that unit was not included in the data analysis. The discharge activity provided an indication for each cell, and the waveforms provided a tracker for identifying the raw signals of each electrode. The CHT firing rates were set as the baseline firing rates for each cell. The mean firing rates during the CHT of each set were calculated and compared using a *t*-test (*p* < 0.05). The cells that showed a significant variation in their activity during the CHT of different sets within one block were also not included.

### 2.6. Statistical Analysis

Task-related units needed to be identified for further analysis. If the average firing rates within any of the last three epochs in the RTc, MT, or THT were 2 SDs greater than baseline, the epoch was defined as a task-related unit. Then, each MT was separated into two epochs for analysis purposes: the perturbation delay (PD) and the perturbation response (PR). The PD is the time between central pad release and perturbation onset, while the PR is the time from perturbation onset to target hit. We computed the firing rates during PR, and a *t*-test was used to determine whether the neuronal discharge frequencies were significantly (*p* < 0.05) altered by the perturbation conditions. Special attention was paid to neurons correlated with the target orientation. The epoch firing rates for task-related cells in the unperturbed trials were classified by movement direction (b = 2) and target orientation (a = 3) and tested with a two-way analysis of variance (ANOVA). Analyses based on the combination of these epochs were performed. The numbers of cells that yielded significant (*p* < 0.05) direction, orientation, and orientation direction interaction effects were also counted. These data allowed us to compare event-related cortical activity across perturbation conditions and target conditions.

### 2.7. Predicting Perturbation Condition

The prediction of the perturbation condition was computed from the neural data using the nonlinear neural network method. We analyzed the data for the left target and the right target separately. A classical two-layer backpropagation neural network was also used to predict perturbation conditions using *n* motor cortical neurons. We represented the responses of multiple neurons by setting *n* input nodes for the network inputs. Hyperbolic tangent sigmoid transfer and linear transfer functions were used among the input, hidden, and output layers. The network consisted of one node representing the predicted perturbation condition. Each data set was randomly partitioned into a training set (1/2 of the data) and a testing set (1/2 of the data), and the weights of each network were then trained so that the sum squared error for the training set was minimized.

## 3. Results

### 3.1. Distribution of 885 Task-Related Motor Cortical Neurons

**Figure 3:**
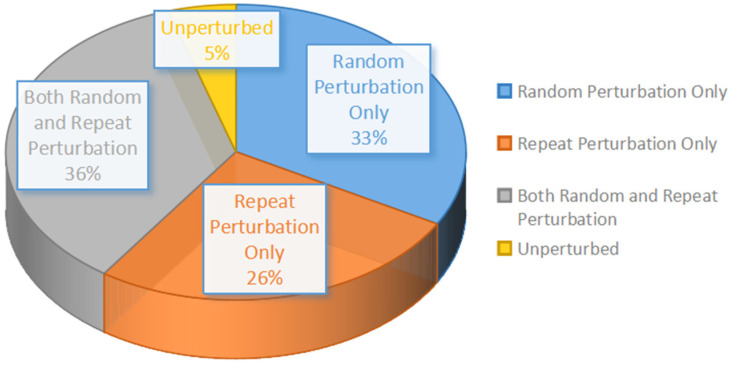
A Pie chart showing the distribution of cortical encoding.

We fully recorded the activity of 1048 motor cortical cells, while 885 out of them were task-related. By summarizing the task-related neuronal coding, we found that 33% of the neurons (293 of 885) were correlated with random perturbation only, 26% (235 of 885) with repeat perturbation only, 36% (315 of 885) with both random and repeat perturbation, and 5% (42 of 885) with unperturbed (Figure 3).

### 3.2. Behavioral Responses to Perturbations

**Figure 4:**
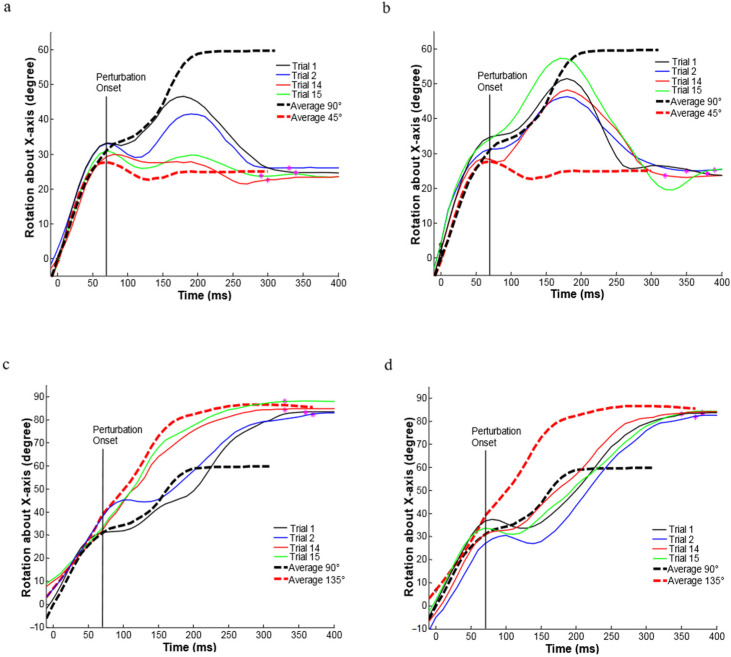
(**a**) Comparison of hand orientation trajectories under the repeat perturbation at 45° CCW (from 90° to 45°). The four solid lines on each plot show four trials (the first two and the last two) for hand orientation trajectories recorded within one set. The two thick dashed lines were averaged from the unperturbed trials recorded in the same block. The magenta stars indicate target hit. (**b**) Comparison of hand orientation trajectories under the random perturbation conditions: perturbed at 45° CCW (from 90° to 45°). (**c**) Comparison of hand orientation trajectories under the repeat perturbation conditions: perturbed at 45° CCW and perturbed at 45° CW (from 90° to 135°). (**d**) Comparison of hand orientation trajectories under the random perturbation conditions: perturbed at 45° CCW and perturbed at 45° CW (from 90° to 135°).

Figure 4 shows the hand orientation trajectories for movements to a right target perturbed at 45° CCW and 45° CW for Monkey 2. For both repeat (Figure 4a,c) and random (Figure 4b,d) perturbation trials, the black vertical lines represent perturbations that occurred 70 ms after central pad release. Under a random perturbation, after the central pad release, the monkey began a movement towards the original 90° target orientation. After about a 100 ms delay after the perturbation onset, the monkey began a corrective movement to bring the hand into alignment with the new target orientation. The final hand orientation in the perturbation trials was about the same as the final hand orientation reached in the unperturbed trials for the same final target orientation. Similar hand orientation trajectories were observed in the first two repeat perturbation trials (Figure 4a,c); however, the over-rotation decreased, and the correction started earlier in these trials. The hand orientation trajectories in the last two trials shifted towards the trajectory required for the final target orientation, and the movement durations also decreased. No clear orientation trajectory adaptation was observed in the random perturbation trials (Figure 4b,d).

**Figure 5:**
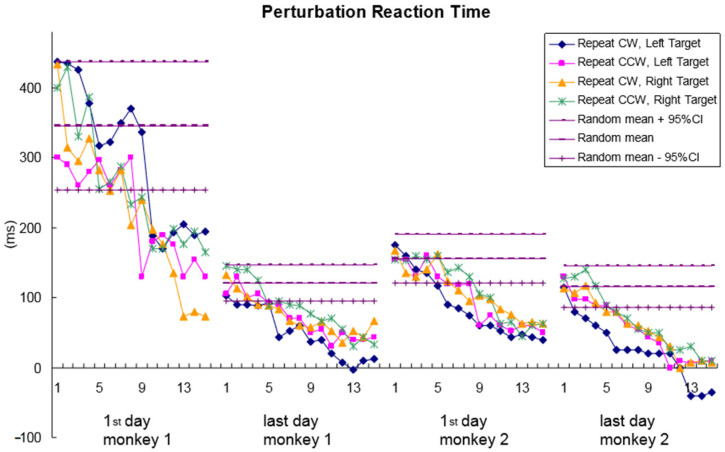
Perturbation reaction time. Mean RTp for different perturbation conditions across consecutive trials on the first and the last perturbation days for the two monkeys. There are seven data lines for each day. The three horizontal lines show the mean RTp (middle) for all the random perturbation trials recorded on that day with 95% confidence intervals (top and bottom lines). Each curved line shows the mean RTp across 15 consecutive trials for one repeat perturbation condition. Each data point was averaged from the RTp of the same trial for the same condition in different blocks recorded on that day.

Figure 5 shows the mean perturbation reaction time (RTp) across consecutive trials for each perturbed condition recorded on the first and the last perturbation days for the two monkeys. There are seven data lines for each day in the figure. The three horizontal lines for the perturbation reaction time show the mean RTp (middle) for all of the random perturbation trials recorded on that day, with a 95% confidence interval (top and bottom lines). The four curved lines show the average RTp across 15 consecutive trials for the four different repeat perturbation conditions. Each data point was averaged from the RTp of the same trial for the same condition in different blocks recorded on that day. An RTp less than the 95% CI lower bound (the lower line) is an indication of the animal predicting the perturbation. As shown in this figure, for all of the four repeat perturbation conditions, both monkeys were able to predict the perturbation after a few trials, although it took them a few more trials on the first perturbation day before starting to predict. On the last perturbation day, the monkey had predicted in only one trial for some repeat perturbation conditions. For some conditions, the RTp was close to or even less than zero, meaning that the hand orientation trajectories were detectably different from the mean trajectory to the vertical target prior to the perturbation onset.

### 3.3. Neuronal Responses to Perturbations

**Figure 6:**
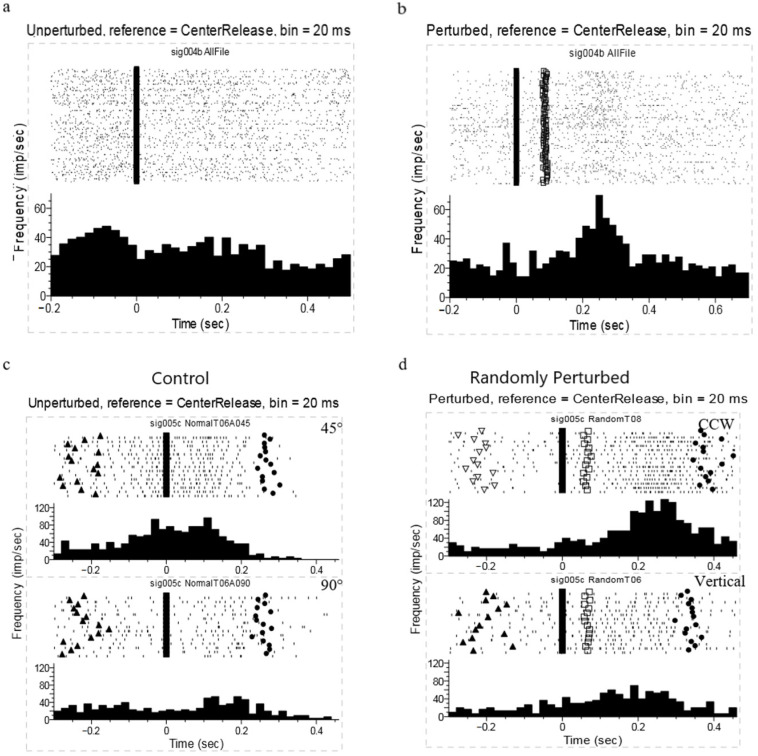
Perievent raster plots for perturbation conditions in comparison. (**a**) Perievent raster histograms for a single unit during 90-degree unperturbed trials. Time zero was aligned to the central pad release. (**b**) Perievent raster histograms for a single unit during 90-degree random perturbation trials. Time zero was aligned to the central pad release. Perturbation occurred at 70 ms after center release (squares). The histograms were calculated with a bin of 20 ms. (**c**) Perievent rasters for a single unit during the unperturbed trials. Time zero was aligned to movement onset. The top plot shows the unperturbed trials at 45 degrees; the bottom shows the unperturbed trials recorded at 90 degrees. The triangles represent target light on, and the circles represent target hit. (**d**) Perievent rasters for a single unit during the random perturbation trials. The top plot shows the random CCW perturbation trials; the right-bottom plot shows the unperturbed trials recorded within the random perturbation set. Time zero was aligned to movement onset. Perturbation occurred at 70 ms after center release (squares). The triangles represent target light on, and the circles represent target hit. (**a**,**b**) originate from the same neuron (sig004b), (**c**,**d**) originate from the another neuron (sig005c).

The raster plots and histograms (see Figure 6a,b) demonstrate a single-unit spiking activity in unperturbed and random perturbation trials (all the trials for both targets). The timings for each spike in each trial were referenced to the central pad release. In the unperturbed trials, this unit started to fire about 200 ms before the center release; it remained active during the movements and then gradually faded out through the trials. In random perturbation, the neuron started to fire until about 50 ms after the center release, and there was an enormous excitatory reaction after the perturbation, peaking nearly 250 ms after the center release.

The perievent histograms (see Figure 6c,d) illustrate the spike activity of an orientation-related neuron during the unperturbed (left) and the random perturbation (right) trials. The spike times for each trial were referenced to the central pad release. In the unperturbed trials, the neuronal activity for the 45° target was much higher than that for the 90° target movements. The neuron fired around 100 ms before the center release during movements to the 45° target, and the movement duration was around 270 ms. Under random 45° CCW perturbation, the neural activity before perturbation onset was much smaller than that during movements to the fixed 45° target but similar to the cellular activity in 90° target movements (both control and random perturbation). After the perturbation onset, there was an enormous excitatory reaction after perturbation, peaking approximately 250 ms after the center release. The movement duration increased to about 400 ms. The huge activity after perturbation was not observed in the unperturbed trials recorded within the random perturbation set, which suggested that the huge activity was probably related to the voluntary motor correction of the hand orientation after perturbation. Our repeat perturbation was highly predictable, and the kinematic results show that the animals were capable of perturbation prediction in a few trials. Sometimes, the RTp was even less than zero, suggesting they were planning an action for a target orientation not actually present. If the animals developed an appropriate predictive strategy, we might expect to see changes in the neural activity before the perturbation onset.

**Figure 7:**
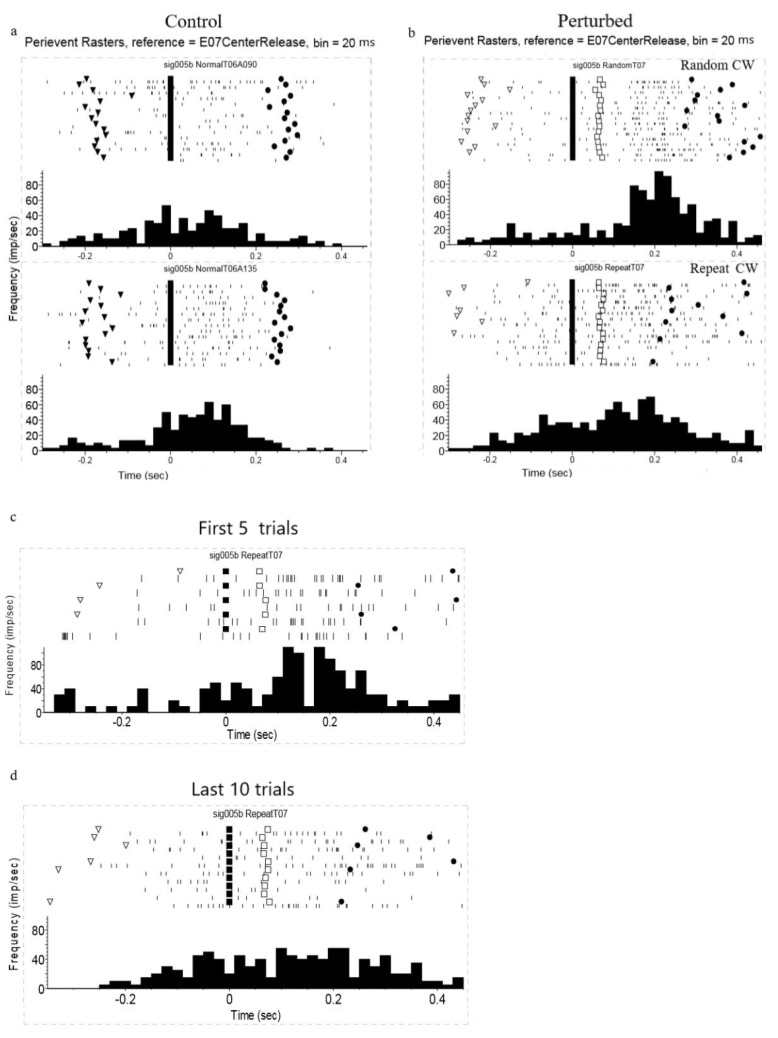
Perievent rasters for single unit in different perturbation conditions. (**a**) Perievent rasters for single unit during the unperturbed trials. Time zero was aligned to movement onset. The triangles represent target light on, and the diamonds represent target hit. (**b**) Perievent raster plot for the single unit during the unperturbed and perturbation trials. The top plot shows the random CW perturbation trials; the bottom shows the repeated CW perturbation trials. Time zero was aligned to movement onset. Perturbation occurred at 70 ms after center release (squares). The triangles represent target light on, and the diamonds represent target hit. (**c**) Perievent raster plot for the single unit during the first five repeated perturbation (bottom) trials. Time zero was aligned to movement onset. Perturbation occurred at 70 ms after center release (squares). The triangles represent target light on, and the diamonds represent target hit. (**d**) Perievent raster plot for the single unit during the last ten repeated perturbation (bottom) trials. Time zero was aligned to movement onset. Perturbation occurred at 70 ms after center release (squares). The triangles represent target light on, and the diamonds represent target hit. All data originate from the same neuron (sig005b) in different tasks.

Figure 7a,b compare the spike activity of a single neuron under different perturbation conditions. Great firing activity was observed 100–200 ms after perturbation onset for the random perturbation. Lower activity after perturbation was found under the repeat perturbation, and the cell’s firing rate before the perturbation onset was higher; this was probably related to the anticipation of the perturbation. The neural spike data for this single unit during the first five and last 10 repeat perturbation trials were also plotted separately for comparison (see Figure 7c). Huge firing activity after perturbation was observed during the first five repeat perturbation trials, which was quite similar to that observed in the random perturbation trials. While this huge activity after perturbation was not observed during the last ten repeat perturbation trials, this transition observed from the neural spike data for the early and late trials was compatible with the observed improvement in the monkeys’ movement kinematics.

**Figure 8:**
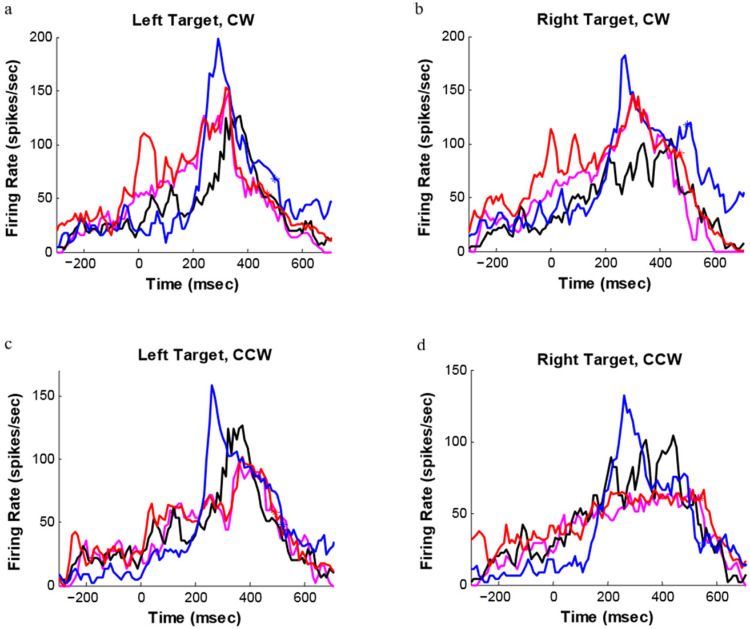
Histograms for one motor cortical neuron during movements (**a**) to left targets under CW perturbation with repeat perturbation (red), with random perturbation (blue), 90° unperturbed (black), and 135° unperturbed (magenta); (**b**) to right targets under CW perturbation with repeat perturbation (red), with random perturbation (blue), 90° unperturbed (black), and 135° unperturbed (magenta); (**c**) to left targets under CCW perturbation with repeat perturbation (red), with random perturbation (blue), 90° unperturbed (black), and 45° unperturbed (magenta); (**d**) to right targets under CCW perturbation with repeat perturbation (red), with random perturbation (blue), 90° unperturbed (black), and 45° unperturbed (magenta).

Figure 8 shows the histograms for an orientation-related-only neuron during movements to the two targets under CW (A and B) and CCW (C and D) perturbations. The black curves represent histograms for movements to 90° unperturbed targets (consistent with the initial target orientation perturbation trials). The magenta curves are histograms for movements to targets fixed at 135° (A and B) and 45° (C and D) in the unperturbed trials (corresponding to the final target orientation perturbation trials). Huge perturbation responses were found around 150 ms after perturbation onset under all of the four random perturbation conditions. A significant increase in neural activity before the perturbation onset under the repeat perturbation conditions was found, and its discharge pattern after perturbation onset matched that observed for the unperturbed final orientation trials.

### 3.4. Prediction of Perturbation Conditions

**Figure 9:**
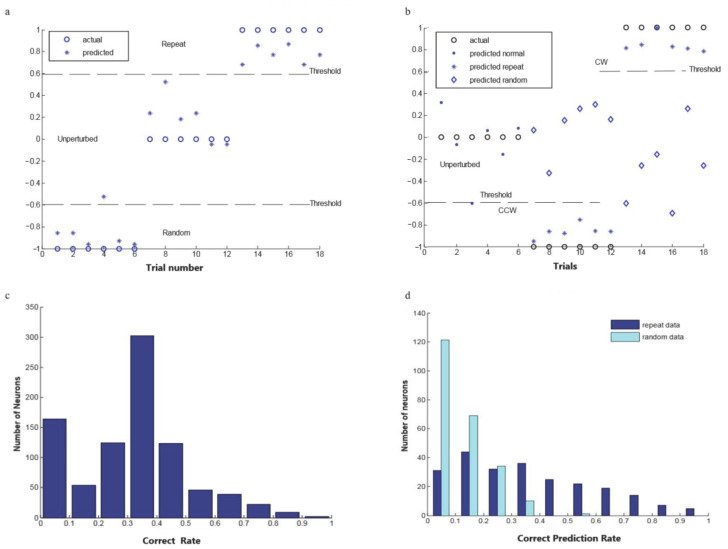
BP network predictions and correct rates. (**a**) Perturbation condition classification results for a typical motor cortical unit using the backpropagation neural network. The *x*-axis shows the trial number. There were 18 testing trials in total (Trial 1~6: actually from random perturbation; Trial 7~12: actually from 90° unperturbed; Trial 13~18: actually from repeat perturbation). (**b**) Repeat perturbation prediction results from a typical motor cortical unit using the backpropagation neural network. The *x*-axis shows the trial number. There were 18 testing trials in total (Trial 1~6: actually from 90° unperturbed; Trial 7~12: actually from repeat 45° CCW; Trial 13~18: actually from repeat 45° CW). (**c**) Distribution of single-unit perturbation condition classification performance. This figure includes 885 task-related neurons recorded during the perturbation experiments. (**d**) Comparison of the perturbation prediction performance between the random data and the repeat data input. This figure only includes neurons that showed significant anticipatory activity (*n* = 235).

Figure 9a shows the perturbation condition classification results from a typical motor cortical unit using the backpropagation neural network. The output values are [−1, 0, 1] (−1 representing random perturbation, 0 representing unperturbed, and 1 representing repeat perturbation). If we set the threshold at 60% (−1~−0.6 for random perturbation, and 0.6~1 for repeat perturbation), this neuron was able to classify the three perturbation conditions with a correct prediction rate of 94.4%. This indicated that this single motor cortical unit was able to classify the repeat perturbation condition from the unperturbed and the randomly perturbed conditions. Then, for all the 885 task-related neurons recorded during the perturbation experiments, we calculated the correct perturbation condition classification rate for each neuron. The threshold was set at 60%, as shown in Figure 9a.

Figure 9b shows the repeat perturbation prediction results from a typical motor cortical unit using the backpropagation neural network. The output values are [−1, 0, 1] (−1 representing repeat perturbation 45° CCW, 0 representing 90° unperturbed, and 1 representing repeat perturbation 45° CW). If we set the threshold at 60% (−1~−0.6 for repeat perturbation 45° CCW, and 0.6~1 for repeat perturbation 45° CW), this neuron had a correct prediction rate of 94.4%. The random perturbation data were also used to test the network, and the results showed that the random data were not able to predict the repeat perturbation. The correct prediction rate for the random data for this neuron was 0. Figure 9b shows that this single neuron was able to predict the two repeat perturbation conditions, and it was also able to classify the repeat perturbation condition from the unperturbed and the random perturbation conditions. Figure 9c shows the distribution of their classification performance for the perturbation conditions. This figure indicates that there were three clusters of neurons. One group had a very low correct prediction rate (0~0.2), and these neurons did not actually show much difference under the three different perturbation conditions. Another group had a fair correct prediction rate (0.2~0.5), and these neurons were mostly from those that had a significant corrective activity but lacked anticipatory activity. They were able to classify the perturbation trials from the unperturbed trials, but they could not classify between the random and the repeat perturbations. The other group had a relatively high correct prediction rate (0.5~1), and these neurons were mostly from those that had both significant correction activity and significant anticipatory activity. They were not only able to classify the perturbation trials from the unperturbed trials but also able to classify between the random and the repeat perturbation conditions. For all the 235 neurons that showed significant anticipatory activity, their correct prediction rate was compared for the results generated from the repeat data and the random data. The threshold was set at 60% (−1~−0.6 for repeat perturbation 45° CCW, and 0.6~1 for repeat perturbation 45° CW). Figure 9d shows the comparison of their prediction performance distribution. The average correct prediction rate for the repeat data appears to be higher than that for the random data. Most neurons had a correct prediction rate of less than 40% under the random perturbation, while about half the number of neurons had a correct prediction rate greater than that under the repeat perturbation.

## 4. Discussion

Our work investigated the discharge patterns of a large number of neurons in the region of the primary motor cortex corresponding to hand and arm movements during perturbation reach-and-grasp trials. Four major observations are reported. First, 69% (608 of 885) of the task-related neurons showed significant voluntary motor correction activity under random perturbation. Second, similar voluntary motor correction activity was found in about half the number of these motor cortical neurons under repeat perturbation but with a smaller magnitude and shorter delay. Third, 27% (235 of 885) of the task-related neurons showed preparatory activity in anticipation of the perturbation. Fourth, the corresponding hand orientation trajectory adaptation and reaching velocity profile adaptation were also observed during repeated perturbation trials. Significant voluntary motor correction activity was found in 608 motor cortical neurons under the random target orientation perturbation. Among them, 315 neurons also showed significant voluntary motor correction activity under the repeat perturbation, but with a lower gain (magnitude), and their activity after perturbation peaked earlier. A total of 235 neurons were also found to have increased activity preceding the onset of the perturbation. This indicates that those neurons were actually predicting or preparing for the perturbation instead of showing correction activity after perturbation. These results suggest that a large quantity of primary motor cortex neurons are responsible for both the online control and predictive control of hand movement. Such findings expand our knowledge regarding the preparation and execution of the neuronal mechanisms underlying motor control. The hand orientation kinematics and transport velocity were also sympathetic to the perturbation of the target orientation within the prehension actions.

In the perturbation trials, the movement duration was lengthened. Over-rotation (relative to the final orientation) was observed in the central nervous system (in both repeat perturbation trials and random perturbation trials). After perturbation, when the initial target orientation was changed, the animals were able to correct the movements during execution, showing that reaching and grasping movements can be corrected online. Moreover, the final hand orientation for the perturbation trials was the same as that realized when the object was initially oriented along the final orientation during the unperturbed trials.

During the repeat perturbation trials, the target orientation perturbation became a fixture of the task in that the monkeys could anticipate its occurrence. As a result, both animals were able to develop a predictive strategy rather than relying on visual feedback. The kinematic results show that the perturbation reaction time and the over-rotation decreased over the trials as the control system learned the perturbation and adapted to its effect. The hand orientation trajectory shifted to match the unperturbed trajectory for the final orientation. Both monkeys showed prediction after a few trials under all four repeat perturbation conditions. They were aware of the alteration of the target orientation and anticipated the final target orientation, which resulted in a dramatically decreased or negative value for the RTp; this suggests that the animals were planning actions against a target orientation that was not yet present.

In the random perturbation cases, the system could respond to the reformed task contingencies with a suitable latency. However, the animals could not predict the perturbation in the random perturbation trials; their corresponding RTp did not decrease over the trials. Thus, no clear orientation trajectory alteration was taken into consideration. Therefore, they were forced to respond to the perturbations for online correction. Collectively, the results suggest a feed-forward approach to perturbation anticipation and preparation and reduced reliance on visual feedback. We identified correlations between the changes in the behaviors and the recorded neural activity patterns observed. Under repeat perturbation, a significant number of primary motor cortex neurons exhibited predictive increases in activity that preceded the perturbation onset and thereby reduced the relative contribution of online correction. The perturbation might also have caused some encoding property changes in some neurons. The distribution of the cortical encoding of the movement parameters of the 885 task-related neurons under the unperturbed and the repeated perturbation conditions was slightly different. That might also have been caused by the fact that the non-specific perturbation responses were the dominant feature in the unit activity and masked the normal tuning properties of the neurons.

## 5. Conclusions

In conclusion, the current results suggest that when the disturbance information is predictable during movement, subjects are more likely to optimize feed-forward planning, than feedback correction. Online feedback control is mostly responsible for perfecting movement execution. In such models, the online feedback trajectory control must be visually guided. It requires the formation of a movement plan and the selection of the target from the visual scene. In addition, these models also play an ongoing role throughout the whole movement [19,20]. The feedback system, in some cases, behaves as if it were two separate systems. The slow mechanism case exhibits cognitive control, and the other case shows fast visual feedback control [21]. The neural control system was given the opportunity to alter the control strategy and update the internal model for a predictable perturbation, while an unpredictable perturbation prevented adaptation and forced the CNS to shift its control emphasis to utilize feedback control. There is the possibility of both types of mechanisms co-existing in the CNS. Ideally, a feed-forward strategy would be likely to be adopted when the demands of the motor task were known in advance, since the feed-forward model plays a dominant role in producing accurate control of movement. However, the CNS might recruit an online feedback control mechanism as the difficulty of the task increases. In addition to task difficulty, mechanism recruitment is also influenced by task familiarity and the priorities of multiple competing performance goals as well as the environment.

However, a small sample size is one of the shortcomings of this experiment. We only used two monkeys in the study, which was really too small a sample size and may affect the homogeneity of results. For instance, there is a large difference in perturbation RTs at the beginning between the two monkeys, which contributes to different looking curves and more disperse results at the end (Figure 5). From the above consideration, it is of great value to keep caution in interpreting and generalizing the findings. In addition, characteristics of the neurons recorded were not specified completely, such as types of neurons, because of the limitation of our experimental technology. So that invaluable information of single-cell records may not be fully exploited. In future work, we will try to limit the variability on the basis of including additional animals and to explore more value of neuronal coding data using a variety of neurobiological techniques.

## Figures and Tables

**Table 1:** Target orientation conditions in the perturbation experiments.

Set	Trial Number	Target 1	Target 2	Conditions
1	90 trials (15 for each target condition)	45°, 90°, 135° Fixed	45°, 90°, 135° Fixed	Unperturbed
2	90 trials (15 for each target condition)	45° CW, 45° CCW, 90°	45° CW, 45° CCW, 90°	Randomly Perturbed
3	30 trials (15 for each target condition)	45° CW	45° CW	Repeatedly Perturbed
4	30 trials (15 for each target condition)	45° CCW	45° CCW	Repeatedly Perturbed

## Data Availability

Not applicable.
